# Association between heavy metals, high-sensitivity C-reaction protein and 10-year risk of cardiovascular diseases among adult Korean population

**DOI:** 10.1038/s41598-021-94158-9

**Published:** 2021-07-19

**Authors:** Hai Duc Nguyen, Hojin Oh, Ngoc Hong Minh Hoang, Min-Sun Kim

**Affiliations:** Department of Pharmacy, College of Pharmacy and Research Institute of Life and Pharmaceutical Sciences, Sunchon National University, Sunchon, Jeonnam 57922 Republic of Korea

**Keywords:** Environmental sciences, Cardiology

## Abstract

The prevalence of cardiovascular diseases (CVDs) in Korea tends to be increasing. It has worsened during the COVID-19 pandemic. Increasing evidence shows heavy metals are associated with increased CVD risk. We aimed to determine the association between the serum heavy metal levels and 10-year risk of CVDs and to predict risks of CVDs based on marginal effects. Heavy metals were measured by a graphite furnace atomic absorption spectrometry and direct mercury analyzer. The results show a significant relationship between the increase in cadmium, lead, mercury, hs-CRP levels and the 10-year risk of CVD after adjustment for serum cotinine, age group, sex, body mass index, a family history of CVDs, diabetes or hyperlipidemia, high-risk drinking, physical activity, and diabetes. A doubling of serum cadmium, lead, mercury, and hs-CRP was associated with the increase in the 10-year risk of CVD by 0.14%, 0.10%, 0.11% and 0.22%, respectively. Therefore, a special concern should be given to the harmful impacts of heavy metals on the 10-year risk of CVD. It is important to develop a prevention strategy targeting the high-risk population to slow down this progression to risk factors related to heavy metals and reduce prevalence. Remarkedly, hs-CRP is the most validated and widely used inflammatory marker, and could be a potential clinical value in predicting and monitoring CVDs.

## Introduction

Cardiovascular diseases (CVDs), including heart disease (i.e., myocardial infarction, angina, and heart failure) and cerebrovascular disease, are a leading cause of death worldwide^1^. In South Korea, CVDs are one in every five deaths^2^.

Over the past few decades, rapid changes in lifestyles and diets such as (smoking, alcohol consumption, consumption of high–unsaturated fat diets, or low energy diets) have led to a rapid increase in the prevalence of non-communicable diseases (NCDs), especially CVDs in South Korea^3^. Furthermore, inherited DNA sequence variants are known to contribute to the conferring of risk for CVDs^4^; however, the effects of heavy metals on the risk factors of CVDs are also important^5–8^.

Rapid industrialization, urbanization and economic growth have increased heavy metal exposure^9^. Lead exposure is due to gasoline, cigarette smoke, manufacturing processes, and domestic lead-based paints^10,11^. Cadmium exposure can be attributed to cigarette smoke and contaminated food or water^12^, whereas contaminated seafood (e.g., fish, shellfish) is primary source of mercury^13^. Accumulated mercury in organs is associated with the development of carotid atherosclerosis^14^, whereas lead and cadmium may disturb blood clotting and increase the risk of CVDs^5,14^. Furthermore, heavy metals catalyzes the production of reactive oxygen species (ROS) and induces inflammatory mediators leading to damage to endothelial vascular cells^6,15^.

Prevention and management have become a top priority due to the increased global burden of CVDs, especially in the situation of the Coronavirus disease-19 (COVID-19) pandemic^16^. Therefore, the cardiovascular risk assessment should be carried out to classify people who most likely benefit from primary prevention. The Framingham Risk Score recommended by the National Cholesterol Education Program (Adult Treatment Panel III) is the most common assessment tool to evaluate a 10-year risk of CVD^17^. However, few studies have explored the correlations between serum heavy metal levels and risk of CVDs among the adult population with or without diabetes on national scales. In this study, we present evidence that increased serum heavy metal levels are related to increased development of the 10-year risk of CVD among the Korean population. We also show the inflammatory marker, which could be a potential clinical value in predicting and monitoring CVDs.

## Results

9602 participants (mean age 47.3 ± 15.2, min–max: 20–79) that participated in the KNANES 2009–2103, 2016–2017 surveys were included. Table 1 shows baseline characteristics according to gender. Males were significantly more likely to be smokers, unmarried, have high-risk drinking.

**Table 1:** Demographic distribution of participants in Korea from 2009 to 2017.

	Males n = 4724	Females n = 4878
**Demographic and social characteristics**		
Age (year)	47.76 ± 15.25	46.87 ± 15.16
Age group (%)		
20–34	1162 (24.6)	1121 (23.0)
35–39	528 (11.2)	522 (10.7)
40–44	513 (10.9)	448 (9.2)
45–49	462 (9.8)	511 (10.5)
50–54	467 (9.9)	511 (10.5)
55–59	489 (10.4)	543 (11.1)
60–64	398 (8.4)	414 (8.5)
65–69	349 (7.3)	409 (8.4)
70–74	218 (4.6)	235 (4.7)
75–79	138 (2.9)	164 (3.4)
Marital status (%)		
Married	3694 (78.2)	4148 (85.0)
Living alone	1030 (21.8)	730 (15.0)
Residential areas (%)		
Urban	3812 (80.7)	3961 (81.2)
Rural	912 (19.3)	917 (18.8)
Occupation (%)		
Managers, professional	756 (16.0)	578 (11.9)
Office worker, clerical workers	602 (12.7)	398 (8.2)
Service workers, sales workers	549 (11.6)	710 (14.6)
Agriculture, forestry and fishing workers	325 (6.9)	221 (4.5)
Craft, plant and machine operators and assemblers	995 (21.1)	136 (2.8)
Elementary occupations	363 (7.7)	446 (9.0)
Unemployed	1134 (24.0)	2300 (49.0)
Education level (%)		
≤ Middle school	1076 (23.2)	1706 (35.6)
High school	1743 (37.6)	1590 (33.2)
≥ College	1816 (39.2)	1495 (31.2)
Monthly household income (%)*		
< 2000	1232 (26.1)	1457 (29.9)
≥ 2000 and < 4000	1581 (33.5)	1530 (31.3)
≥ 4000 and < 6000	1027 (21.7)	975 (20.0)
≥ 6000	884 (18.7)	916 (18.8)
BMI group (%)		
< 18·5	112 (2.4)	242 (5.0)
≥ 18·5 and < 25	2786 (59.3)	3169 (65.0)
≥ 25 and < 30	1622 (34.3)	1232 (25.2)
≥ 30	190 (4.0)	235 (4.8)
Smoking status (%)		
Non/ex-smoker	2412 (50.8)	4562 (94.1)
Current smoker	2340 (49.2)	288 (5.9)
Cotinine verified smokers (%)		
No	2549 (54.0)	4380 (89.8)
Yes	2175 (46.0)	498 (10.2)
High-risk drinking status (%)		
No	3905 (80.1)	4724 (100.0)
Yes	973 (19.9)	0 (0.0)
Physical activity (%)		
Not regular	3341 (70.7)	3426 (70.2)
Regular	1383 (29.3)	1452 (29.8)
Family history of CVDs (%)		
No	3223 (68.2)	3091 (63.4)
Yes	1501 (31.8)	1787 (36.6)
Family history of diabetes (%)		
No	3960 (83.8)	4031 (82.6)
Yes	764 (16.2)	847 (17.4)
Family history of hyperlipidemia (%)		
No	4514 (95.6)	4590 (94.1)
Yes	210 (4.4)	288 (5.9)

*BMI* body mass index (kg/m^2^), *CVDs* Cardiovascular diseases.

*Thousand won.

The average 10-year risk of CVD was 7.36 ± 7.34. The majority of the subjects were identified in the low-risk (67.3%) category, 21.9% at medium risk, and only 11 percent at high risk. Geometric mean serum cadmium, lead, mercury and high-sensitivity C-reaction protein (hs-CRP) levels were 0.97 µg*/L* (95%CI: 0.95–0.97), 2.02 µg*/dL* (1.10) (95%CI: 2.00–2.03), 3.71 µg*/L* (3.52) (95%CI: 3.66–3.75), and 0.72 mg*/L* (95%CI: 0.70–0.75), respectively.

Table 2 shows the Pearson correlation coefficients (r) between the 10-year risk of CVD, cardio-metabolic risk factors, and dietary intake by gender. We found that strong significant correlations were pointed out between the 10-year risk of CVD and age (r = 0.818 for males and r = 0.828 for females); significant positive correlations were noted between 10-year risk of CVD, total cholesterol (r = 0.515), LDL-C (r = 0.540) and systolic blood pressure (r = 0.645) in females.

**Table 2:** Pearson bivariate correlation between the 10-year risk of CVD and cardiometabolic risk factors, dietary intake by sex.

Variables	Male *n* = 4724	Female *n* = 4878
	r	r
Age (year)	0.818	0.828
BMI (Kg/m^2^)	0.137	0.374
Waist circumference (cm)	0.271	0.456
Total cholesterol (mg/dL)	0.357	0.515
LDL-C (mg/dL)	0.306	0.540
Triglyceride (mg/dL)	0.248	0.376
HDL-C (mg/dL)	− 0.207	− 0.255
HbA1c (%)	0.301	0.364
Fasting glucose (mg/dL)	0.288	0.296
Energy intake (Kcal)	− 0.127	− 0.101
Hemoglobin (g/dL)	− 0.146	0.114
ALT (U/L)	0.066	0.224
AST (U/L)	0.085	0.264
SBP (mmHg)	0.355	0.645
DBP (mmHg)	0.151	0.421
Serum creatinine (μmol/L)	0.040	0.101
BUN (mmol/L)	0.223	0.335
Serum cotinine (ng/mL)	0.116	0.129
Vitamin B1 (mg)	− 0.093	− 0.010
Vitamin B2 (mg)	− 0.118	− 0.134
Vitamin B3 (mg)	− 0.125	− 0.146
Vitamin C (mg)	− 0.046	− 0.054
Total vitamin A (µg)	0.030	− 0.009
Omega 3 (g)	− 0.019	− 0.067
Omega 6 (g)	− 0.232	− 0.225
Serum Cd (µg/L)	0.288	0.376
Serum Pb (µg/dL)	0.245	0.243
Serum Hg (µg/L)	0.221	0.217
hs-CRP (mg/L)	0.194	0.226

*BUN* blood urea nitrogen, *HDL-C* high-density lipoprotein cholesterol, *ALT* alanine aspartate aminotransferase, *AST* aspartate aminotransferase, *LDL-C* low-density lipoprotein cholesterol, *SBP* systolic blood pressure, *DBP* diastolic blood pressure.

**Figure 1:**
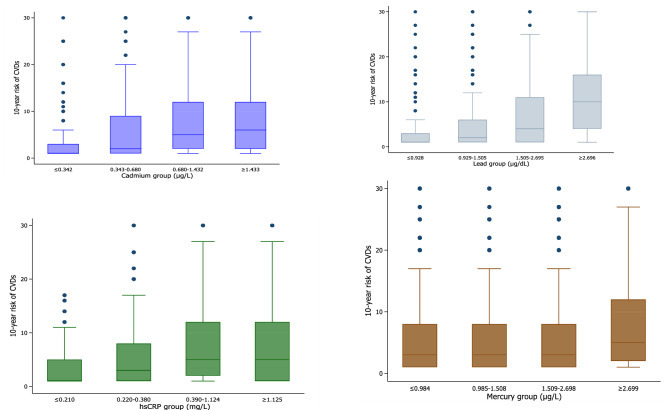
The levels of the 10-year risk of CVD according to the quartiles of serum cadmium, lead, mercury, and hs-CRP among the Korean population.

Figure 1 shows the levels of the 10-year risk of CVD according to the quartiles of serum cadmium, lead, mercury, and hs-CRP among the Korean population. The level of the 10-year risk of CVD was significantly higher among subjects with high serum heavy metal levels or serum hs-CRP levels than those with low serum heavy metal levels or serum hs-CRP levels.

**Figure 2:**
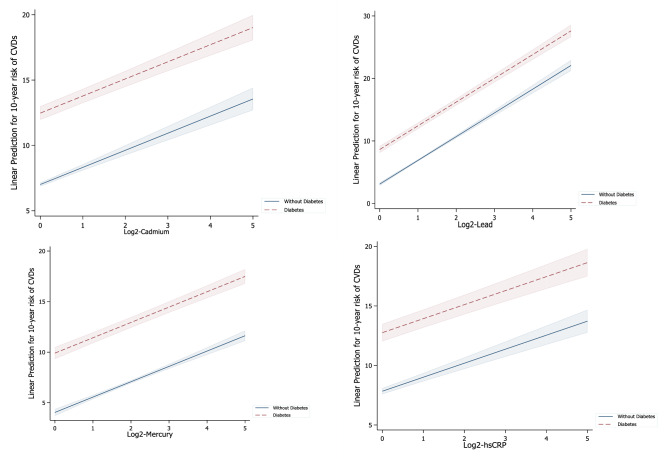
The prediction of 10-year risk of CVD among subjects with or without diabetes by heavy metals and hs-CRP.

Figure 2 shows the prediction of 10-year risk of CVD among subjects with or without diabetes by heavy metals and hs-CRP. A doubling of serum cadmium, lead and mercury was associated with the increase in the 10-year risk of CVD by 5.47% (β = 5.47, 95% CI: 4.93–6.00, p < 0.001), 5.53% (β = 5.53, 95% CI: 5.02–6.04, p < 0.001), and 5.86% (β = 5.86, 95% CI: 5.33–6.40, p < 0.001) among subjects with diabetes, respectively. Similarly, among subjects with diabetes, the 10-year risk of CVD increased by 4.92% (β = 4.92, 95% CI: 4.16–5.68, p < 0.001) with a twofold increase in serum hs-CRP levels.

An increase in serum cadmium, lead, mercury and hs-CRP was associated with an increase in cardiometabolic risk factors in both males and females. As presented in Table 3, age, BMI, waist circumference, total cholesterol, LDL-C, triglyceride, HDL-C, HbA1c, fasting glucose, energy intake, hemoglobin, hematocrit, BUN, creatinine, ALT, AST, systolic blood pressure, and diastolic blood pressure increased significantly among quartiles of serum cadmium, lead, mercury and hs-CRP.

**Table 3:** Cardiometabolic factors according to the quartiles of serum cadmium, lead, mercury, and hs-CRP levels by gender.

Variables	Males (n = 4724)					Females (n = 4878)				
	Cadmium quartiles					Cadmium quartiles				
	Quartile 1 ≤ 0.342 (µg/L)	Quartile 2 0.343–0.680 (µg/L)	Quartile 3 0.680–1.432(µg/L)	Quartile 4 ≥ 1.433 (µg/L)	*P*-value	Quartile 1 ≤ 0.928 (µg/dL)	Quartile 2 0.929–1.505 (µg/dL)	Quartile 3 1.505–2.695 (µg/dL)	Quartile 4 ≥ 2.696 (µg/dL)	*P*-value
**(A) Cardiometabolic factors according to the quartiles of serum cadmium levels by gender**										
Age (year)	32.06 ± 11.58	41.28 ± 14.54	49.38 ± 14.48	52.18 ± 13.44	< 0.001	29.55 ± 9.93	35.61 ± 13.06	48.53 ± 14.47	54.89 ± 12.26	< 0.001
BMI (Kg/m^2^)	24.21 ± 3.59	24.34 ± 3.19	24.37 ± 3.13	24.03 ± 3.06	0.046	22.08 ± 4.15	22.42 ± 3.70	23.54 ± 3.52	24.15 ± 3.46	< 0.001
Waist circumference (cm)	83.48 ± 9.65	84.58 ± 8.89	85.73 ± 8.77	85.57 ± 8.68	< 0.001	73.89 ± 10.32	75.01 ± 10.10	78.83 ± 9.69	81.21 ± 9.55	< 0.001
Total cholesterol (mg/dL)	179.62 ± 34.55	186.97 ± 36.41	190.90 ± 36.73	195.36 ± 38.42	< 0.001	185.81 ± 34.92	187.16 ± 35.75	190.50 ± 37.72	194.83 ± 37.25	< 0.001
LDL-C (mg/dL)	107.32 ± 28.22	114.27 ± 31.32	114.19 ± 31.59	113.91 ± 35.35	0.042	99.23 ± 31.47	104.48 ± 30.12	114.14 ± 32.82	118.71 ± 33.22	< 0.001
Triglyceride (mg/dL) †	99.5 (44–160)	115 (48–174)	131 (53–199.5)	147 (54–216)	< 0.001	68 (33–99)	79 (38–116)	94 (41–141)	106 (46–158)	< 0.001
HDL-C (mg/dL)	47.41 ± 10.33	47.26 ± 10.70	46.18 ± 11.58	46.56 ± 12.39	0.038	56.57 ± 14.03	54.97 ± 12.33	52.99 ± 12.36	51.31 ± 12.36	< 0.001
HbA1c (%)	5.43 ± 0.66	5.72 ± 1.03	5.90 ± 1.04	6.02 ± 1.04	< 0.001	5.49 ± 0.99	5.49 ± 0.80	5.73 ± 0.80	5.85 ± 0.85	< 0.001
Fasting glucose (mg/dL)	92.89 ± 17.39	99.28 ± 25.67	102.68 ± 26.10	104.24 ± 28.28	< 0.001	91.97 ± 20.84	92.82 ± 19.44	96.58 ± 20.29	99.14 ± 22.21	< 0.001
Energy intake (Kcal)	2446.97 ± 1116.32	2428.25 ± 987.62	2362.39 ± 939.62	2347.15 ± 961.58	0.180	1706.65 ± 633.06	1764.11 ± 719.82	1715.66 ± 700.93	1655.23 ± 637.53	0.0041
Hemoglobin (g/dL)	15.34 ± 1.00	15.23 ± 1.08	15.29 ± 1.16	15.39 ± 1.36	0.027	13.09 ± 1.55	13.04 ± 1.60	13.03 ± 1.54	13.12 ± 1.29	0.054
Hematocrit (%)	45.74 ± 2.98	45.43 ± 3.09	45.54 ± 3.33	45.54 ± 3.33	0.117	39.87 ± 2.63	39.78 ± 2.83	39.81 ± 3.01	40.16 ± 3.43	0.004
BUN (mmol/L)	14.04 ± 3.46	14.63 ± 4.22	14.93 ± 4.15	15.05 ± 4.62	0.001	11.90 ± 3.45	12.11 ± 3.52	13.43 ± 3.97	14.14 ± 4.20	< 0.001
Serum creatinine (μmol/L)	0.99 ± 0.43	0.98 ± 0.23	0.96 ± 0.17	0.97 ± 0.26	0.017	0.69 ± 0.09	0.70 ± 0.10	0.71 ± 0.11	0.71 ± 0.16	0.002
ALT (U/L) †	18 (10–27)	21 (11–29)	22 (11–32)	24 (13–35)	< 0.001	11 (6–15)	12 (7–17)	15 (8–20)	16 (9–22)	< 0.001
AST (U/L) †	20 (13–23)	21 (14–25)	22 (15–27)	24 (16–31)	< 0.001	16 (12–19)	16.5 (12–19)	19 (13–27)	20 (14–24)	< 0.001
SBP (mmHg)	116.09 ± 12.13	119.05 ± 14.06	122.06 ± 15.09	124.31 ± 16.46	< 0.001	106.54 ± 11.88	107.95 ± 12.60	116.14 ± 17.09	121.82 ± 18.22	< 0.001
DBP (mmHg)	77.07 ± 8.77	79.00 ± 10.01	79.89 ± 10.27	80.42 ± 11.61	< 0.001	69.66 ± 8.58	70.96 ± 8.80	74.21 ± 9.37	76.75 ± 10.10	< 0.001
Serum cotinine (ng/mL)†	2.27 (0.19–10.51)	3.76 (0.18–69.97)	29.73 (0.24–1200.65)	1156.65 (0.45–1961.12)	< 0.001	1.59 (0.09–8.24)	1.29 (0.08–5.92)	1.4 (0.04–6.73)	1.60 (0.01–9.71)	< 0.001

Variables	Males (n = 4724)					Females (*n* = *4878 )*				
	Mercury quartiles					Mercury quartiles				
	Quartile 1 ≤ 0.984 (µg/L)	Quartile 2 0.985- 1.508 (µg/L)	Quartile 3 1.509–2.698 (µg/L)	Quartile 4 ≥ 2.699 (µg/L)	p-value	Quartile 1 ≤ 0.984 (µg/L)	Quartile 2 0.985- 1.508 (µg/L)	Quartile 3 1.509–2.698 (µg/L)	Quartile 4 ≥ 2.699 (µg/L)	p-value
**(B) Cardiometabolic factors according to the quartiles of serum mercury levels by gender**										
Age (year)	54.40 ± 19.90	45.02 ± 19.86	45.64 ± 17.73	47.11 ± 14.24	0.0003	50.74 ± 19.84	48.47 ± 17.15	46.61 ± 15.71	48.20 ± 14.53	0.001
BMI (Kg/m^2^)	22.79 ± 3.62	23.50 ± 3.61	23.51 ± 3.25	24.50 ± 3.09	< 0.001	22.74 ± 3.83	22.78 ± 3.50	23.25 ± 3.56	23.74 ± 3.59	< 0.001
Waist circumference (cm)	81.85 ± 9.98	83.41 ± 10.30	83.23 ± 9.23	85.82 ± 8.63	< 0.001	78.13 ± 11.15	77.19 ± 10.05	78.06 ± 10.03	79.38 ± 9.88	< 0.001
Total cholesterol (mg/dL)	181.60 ± 30.00	178.71 ± 34.39	181.59 ± 35.84	192.01 ± 36.89	< 0.001	185.06 ± 34.71	188.02 ± 38.07	190.10 ± 38.64	193.14 ± 36.66	0.004
LDL-C (mg/dL)	120.28 ± 35.37	106.10 ± 27.70	108.94 ± 31.18	114.84 ± 32.39	0.0002	103.26 ± 30.68	111.66 ± 31.06	112.94 ± 34.94	114.34 ± 32.07	0.151
Triglyceride (mg/dL) †	114.5 (45–149)	121 (51–184)	113 (49–170)	133 (52–201)	< 0.001	86 (44–158)	99 (41–138)	94 (40–140)	95 (40–141)	0.607
HDL-C (mg/dL)	44.93 ± 11.47	46.25 ± 12.95	46.57 ± 11.45	46.62 ± 11.41	0.790	53.73 ± 15.10	52.54 ± 12.65	52.64 ± 12.52	53.07 ± 12.37	0.590
HbA1c (%)	5.85 ± 0.92	5.73 ± 1.01	5.80 ± 1.07	5.87 ± 1.02	0.285	5.66 ± 0.77	5.76 ± 0.97	5.66 ± 0.77	5.76 ± 0.84	0.031
Fasting glucose (mg/dL)	98.10 ± 19.92	101.88 ± 29.17	99.92 ± 27.26	101.89 ± 25.77	0.220	98.06 ± 21.59	98.01 ± 27.14	95.22 ± 18.54	97.14 ± 21.21	0.013
Energy intake (Kcal)	1784.09 ± 680.97	2213.05 ± 886.77	2267.59 ± 945.03	2419.37 ± 973.98	< 0.001	1504.31 ± 573.10	1639.54 ± 633.07	1723.52 ± 692.11	1707.36 ± 686.35	0.013
Hemoglobin (g/dL)	14.51 ± 1.61	14.94 ± 1.41	15.04 ± 1.28	15.37 ± 1.12	< 0.001	12.46 ± 1.63	12.75 ± 1.28	12.90 ± 1.15	13.21 ± 1.05	< 0.001
Hematocrit (%)	43.93 ± 4.37	44.70 ± 3.78	44.99 ± 3.68	45.74 ± 3.20	< 0.001	38.90 ± 4.40	39.27 ± 3.38	39.43 ± 3.13	40.29 ± 2.96	< 0.001
BUN (mmol/L)	14.36 ± 4.25	13.71 ± 4.55	14.54 ± 5.30	14.93 ± 3.94	0.0006	13.63 ± 4.90	13.04 ± 3.95	13.01 ± 4.09	13.62 ± 3.95	< 0.001
Serum creatinine (μmol/L)	0.98 ± 0.18	0.99 ± 0.24	0.99 ± 0.41	0.96 ± 0.17	0.004	0.78 ± 0.22	0.71 ± 0.12	0.71 ± 0.12	0.70 ± 0.13	< 0.001
ALT (U/L) †	16.5 (10–23)	18 (9–26)	19 (10–27)	23 (11–33)	< 0.001	13 (8–17)	14 (7–19)	14 (8–19)	15 (8–21)	< 0.001
AST (U/L) †	20 (15–25)	20 (13–25)	20 (13–25)	22 (15–27)	0.076	19 (13–21)	18 (12–23)	18 (13–22)	19 (13–23)	< 0.001
SBP (mmHg)	123.45 ± 16.77	120.46 ± 15.96	119.42 ± 15.47	121.80 ± 14.94	0.001	117.80 ± 19.02	115.98 ± 18.26	114.97 ± 17.14	116.93 ± 17.30	0.003
DBP (mmHg)	74.26 ± 11.68	77.58 ± 10.15	76.86 ± 10.18	80.30 ± 10.36	< 0.001	73.46 ± 9.15	73.10 ± 10.09	73.39 ± 9.44	74.99 ± 9.79	< 0.001
Serum cotinine (ng/mL) †	5.61 (0.21–967)	8.61 (0.21–1108.25)	12.58 (0.24–1180.58)	17.76 (0.25–1170.28)	0.350	0.53 (0.06–2.09)	0.82 (0.12–4.55)	1.29 (0.09–6.27)	1.77 (0.01–8.74)	0.002

Variables	Males (n = 4724)					Females (n = 4878 )				
	Lead quartiles					Lead quartiles				
	Quartile 1 ≤ 0.984 (µg/dL)	Quartile 2 0.984–1.509 (µg/dL)	Quartile 3 1.509–2.698 (µg/dL)	Quartile 4 ≥ 2.699 (µg/dL)	p-values	Quartile 1 ≤ 0.984 (µg/dL)	Quartile 2 0.984–1.509 (µg/dL)	Quartile 3 1.509–2.698 (µg/dL)	Quartile 4 ≥ 2.699 (µg/dL)	p-values
**(C) Cardiometabolic factors according to the quartiles of serum lead levels by gender**										
Age (year)	35.53 ± 15.82	39.46 ± 15.33	45.56 ± 15.24	51.66 ± 13.20	< 0.001	37.35 ± 13.94	44.04 ± 15.53	49.49 ± 14.69	54.93 ± 12.09	< 0.001
BMI (Kg/m^2^)	24.29 ± 3.86	24.37 ± 3.45	24.35 ± 3.22	24.18 ± 2.95	0.343	22.80 ± 3.69	23.16 ± 3.69	23.65 ± 3.51	24.04 ± 3.31	< 0.001
Waist circumference (cm)	84.19 ± 10.61	84.90 ± 9.44	85.32 ± 9.00	85.42 ± 8.38	0.430	76.66 ± 10.96	77.53 ± 10.24	79.29 ± 9.74	80.66 ± 9.29	< 0.001
Total cholesterol (mg/dL)	181.31 ± 30.45	185.89 ± 34.47	189.18 ± 37.61	192.27 ± 36.67	0.002	187.84 ± 35.04	187.69 ± 36.53	191.83 ± 37.63	201.15 ± 38.03	< 0.001
LDL-C (mg/dL)	108.81 ± 29.29	111.76 ± 28.78	113.85 ± 32.57	114.14 ± 32.48	0.558	106.80 ± 31.20	109.00 ± 32.77	113.03 ± 32.50	123.23 ± 32.99	< 0.001
Triglyceride (mg/dL) †	110.5 (37.5–191)	109 (44–168)	126 (52–190)	139 (54–210)	< 0.001	81 (36–122)	88 (40–130)	96 (42–142)	112 (45–164.5)	< 0.001
HDL-C (mg/dL)	46.68 ± 10.92	46.54 ± 10.85	46.28 ± 11.27	47.02 ± 11.95	0.245	55.37 ± 13.07	53.53 ± 12.30	52.67 ± 12.64	51.08 ± 11.64	< 0.001
HbA1c (%)	5.65 ± 1.00	5.71 ± 0.99	5.86 ± 1.06	5.92 ± 0.99	0.0007	5.52 ± 0.79	5.66 ± 0.82	5.77 ± 0.85	5.86 ± 0.79	< 0.001
Fasting glucose (mg/dL)	100.68 ± 31.07	98.85 ± 26.08	101.74 ± 27.49	102.21 ± 23.79	0.055	93.03 ± 19.67	96.01 ± 20.16	97.25 ± 21.26	97.54 ± 21.58	0.001
Energy intake (Kcal)	2151.62 ± 978.46	2385.41 ± 981.32	2369.51 ± 961.48	2404.58 ± 969.45	0.177	1781.22 ± 729.61	1726.66 ± 713.00	1701.55 ± 665.56	1622.86 ± 652.54	0.002
Hemoglobin (g/dL)	15.17 ± 1.25	15.22 ± 1.22	15.29 ± 1.13	15.33 ± 1.21	0.156	12.71 ± 1.24	12.89 ± 1.19	13.13 ± 1.07	13.39 ± 1.04	< 0.001
Hematocrit (%)	45.69 ± 3.61	45.73 ± 3.53	45.62 ± 3.25	45.45 ± 3.37	0.235	39.21 ± 3.33	39.60 ± 3.27	40.01 ± 3.00	40.63 ± 2.90	< 0.001
BUN (mmol/L)	13.95 ± 3.95	14.19 ± 4.18	14.70 ± 4.12	15.25 ± 4.34	< 0.001	11.49 ± 3.42	12.89 ± 3.72	13.59 ± 3.95	14.76 ± 4.61	< 0.001
Serum creatinine (μmol/L)	0.95 ± 0.16	0.97 ± 0.36	0.97 ± 0.20	0.97 ± 0.21	0.976	0.67 ± 0.10	0.70 ± 0.11	0.71 ± 0.11	0.73 ± 0.20	< 0.001
ALT (U/L) †	18 (10–31)	21 (10–33)	22 (11–32)	22 (11–31)	0.699	12 (7–18)	14 (7–19)	15 (8–21)	16 (9–22)	0.009
AST (U/L) †	19.5 (13–24)	21 (13–26)	22 (15–27)	22 (15–28)	0.076	17 (13–20)	18 (13–21)	19 (13–23)	20 (14–24)	< 0.001
SBP (mmHg)	118.25 ± 13.33	118.00 ± 13.33	120.05 ± 14.01	124.46 ± 16.57	< 0.001	109.70 ± 13.95	112.95 ± 16.21	117.55 ± 17.72	121.92 ± 17.72	< 0.001
DBP (mmHg)	75.91 ± 9.48	77.42 ± 9.54	79.20 ± 10.20	81.03 ± 10.85	< 0.001	71.51 ± 8.98	72.51 ± 9.23	74.90 ± 9.76	77.43 ± 9.86	< 0.001
Serum cotinine (ng/mL) †	2.50 (0.27–512.02)	4.39 (0.18–817)	13.20 (0.24–1135.96)	97.82 (0.33–1330.00)	< 0.001	0.674 (0.06–3.08)	0.84 (0.07–4.56)	1.79 (0.01–8.49)	3.15 (0.01–10.83)	0.002

	Males (n = 4724)					Females (n = 4878 )				
	hs-CRP quartiles					hs-CRP quartiles				
	Quartile 1 ≤ 0.21 (µg/L)	Quartile 0.22–0.38 (µg/L)	Quartile 0.39- 1.124 (µg/L)	Quartile 4 ≥ 1.125 (µg/L)	P-values	Quartile 1 ≤ 0.21 (µg/L)	Quartile 0.22–0.38 (µg/L)	Quartile 0.39–1.124 (µg/L)	Quartile 4 ≥ 1.125 (µg/L)	P-values
**(D) Cardiometabolic factors according to the quartiles of serum hs-CRP levels by gender**										
Age (year)	43.39 ± 14.13	46.06 ± 16.43	49.46 ± 15.29	46.23 ± 14.93	< 0.001	43.16 ± 14.68	47.00 ± 15.02	51.62 ± 14.88	46.87 ± 15.21	< 0.001
BMI (Kg/m^2^)	22.34 ± 2.30	23.04 ± 2.77	24.57 ± 2.99	24.35 ± 3.22	< 0.001	21.08 ± 2.56	22.08 ± 2.91	23.99 ± 3.44	23.66 ± 3.67	< 0.001
Waist circumference (cm)	80.54 ± 6.10	81.84 ± 7.64	86.38 ± 7.99	85.34 ± 9.14	< 0.001	72.29 ± 7.15	75.15 ± 8.00	80.47 ± 9.57	79.06 ± 10.25	< 0.001
Total cholesterol (mg/dL)	186.11 ± 31.73	185.89 ± 33.27	192.66 ± 36.35	189.33 ± 37.33	0.016	187.35 ± 30.20	190.47 ± 3386	196.31 ± 39.49	196.31 ± 39.49	0.001
LDL-C (mg/dL)	97.25 ± 47.26	105.53 ± 27.10	114.91 ± 32.74	113.78 ± 32.18	0.277	118.33 ± 38.04	113.13 ± 33.38	115.23 ± 38.34	113.57 ± 32.67	0.931
Triglyceride (mg/dL) †	102 (49–135)	107 (46–152)	134 (53–203)	130 (50–199)	0.001	72 (37–99)	81 (37–112)	105 (47–151)	95 (40–144)	< 0.001
HDL-C (mg/dL)	52.44 ± 10.05	50.79 ± 12.10	47.67 ± 11.95	45.82 ± 11.15	< 0.001	58.79 ± 12.97	58.33 ± 13.31	53.34 ± 12.44	51.73 ± 12.07	< 0.001
HbA1c (%)	5.52 ± 0.91	5.51 ± 0.65	5.71 ± 0.80	6.00 ± 1.17	< 0.001	5.37 ± 0.36	5.49 ± 0.57	5.67 ± 0.73	5.86 ± 0.96	< 0.001
Fasting glucose (mg/dL)	99.14 ± 23.81	98.88 ± 21.61	104.22 ± 24.86	101.03 ± 26.78	0.002	92.30 ± 12.41	95.06 ± 17.90	100.10 ± 22.66	95.94 ± 20.93	< 0.001
Energy intake (Kcal)	2344.50 ± 876.17	2339.90 ± 882.38	2340.94 ± 991.29	2395.93 ± 969.35	0.476	1700.97 ± 676.59	1741.26 ± 694.23	1707.16 ± 718.47	1697.80 ± 671.34	0.673
Hemoglobin (g/dL)	15.16 ± 1.26	15.23 ± 1.13	15.30 ± 1.15	15.30 ± 1.18	0.671	12.91 ± 1.31	12.95 ± 1.14	13.18 ± 1.14	13.05 ± 1.11	0.0004
Hematocrit (%)	46.06 ± 3.27	46.22 ± 3.27	46.57 ± 3.30	45.21 ± 3.29	< 0.001	40.04 ± 3.26	40.25 ± 3.07	40.96 ± 3.19	39.54 ± 3.01	< 0.001
BUN (mmol/L)	15.16 ± 4.30	14.78 ± 3.77	15.01 ± 4.22	14.77 ± 4.26	0.436	12.31 ± 3.36	13.34 ± 3.88	13.81 ± 4.34	13.31 ± 3.96	0.001
Serum creatinine (μmol/L)	1.00 ± 0.28	0.96 ± 0.15	0.96 ± 0.19	0.97 ± 0.24	0.558	0.71 ± 0.10	0.70 ± 0.10	0.71 ± 0.13	0.71 ± 0.13	0.868
ALT (U/L) †	17 (11–24.5)	19 (10–24)	23 (11–33)	22 (11–32)	0.001	13 (8–17)	13 (8–19)	16 (8–21)	15 (8–21)	< 0.001
AST (U/L) †	19 (14–22)	20 (14–24)	22 (15–27)	22 (14–27)	0.007	17 (13–20)	18 (13–22)	19 (13–23)	19 (13–22)	< 0.001
SBP (mmHg)	115.25 ± 12.67	117.29 ± 12.53	121.10 ± 14.43	121.91 ± 15.46	< 0.001	111.22 ± 14.43	112.71 ± 15.65	116.94 ± 17.28	116.77 ± 17.66	< 0.001
DBP (mmHg)	75.84 ± 9.23	77.02 ± 9.33	78.28 ± 10.08	80.26 ± 10.56	< 0.001	71.62 ± 7.58	72.12 ± 8.74	73.87 ± 9.57	74.89 ± 9.91	< 0.001
Serum cotinine (ng/mL) †	0.84 (0.26–278.5)	1.16 (0.21–1836)	1.59 (0.25–1064)	26.91 (0.25–1215.37)	< 0.001	0.56 (0.12–1.00)	0.47 (0.14–0.92)	0.51 (0.14–1.05)	3.21 (0.01–10.36)	0.120

*BUN* blood urea nitrogen, *HDL-C* high-density lipoprotein cholesterol, *ALT* alanine aspartate aminotransferase, *AST* aspartate aminotransferase, *LDL-C* low-density lipoprotein cholesterol, *SBP* systolic blood pressure, *DBP* diastolic blood pressure.

^†^Median (IQR) and p-value using Kruskal Wallis test.

Table 4 demonstrates the results of multiple regression analysis of the 10-year risk of CVD. The results show a significant relationship between the increase in cadmium, lead, mercury and CRP levels and 10-year risk of CVD after adjustment for age group, serum cotinine, sex, body mass index, a family history of CVDs or diabetes or hyperlipidemia, high-risk drinking, physical activity, and diabetes. A doubling of serum cadmium, lead, mercury, and hs-CRP was associated with the increase in the 10-year risk of CVD by 0.14% (β = 0.14, 95% CI: 0.05–0.23, *p* = 0.003), 0.10% (β = 0.10, 95% CI: 0.02–0.21, *p* < 0.001), 0.11% (β = 0.11, 95% CI: 0.04–0.18, p = 0.003) and 0.22% (β = 0.22, 95% CI: 0.16–0.29, *p* < 0.001), respectively.

**Table 4:** The relationship between the levels of serum cadmium, lead, mercury and hs-CRP and 10-year risk of CVD by multiple regression.

Parameters	Cadmium (μg/L)					hs-CRP (mg/L)				
	ß	SE	95% CI		*p*-value	ß	SE	95% CI		*p*-value
		Adjusted R^2^ = 0.773, *p* < 0.001					Adjusted R^2^ = 0.764, *p* < 0.001			
*(A) For cadmium and hs-CRP*										
Serum Cadmium or hs-CRP	0.135	0.046	0.045	0.225	0.003	0.222	0.033	0.157	0.287	< 0.001
Serum cotinine (ng/mL)	0.0002	0.00005	0.00006	0.0003	0.001	0.0003	0.0007	0.0001	0.0004	< 0.001
**Age group (%)**										
20–34	Refer					Refer				
35–39	5.334	0.114	5.110	5.559	< 0.001	5.077	0.154	4.776	5.378	< 0.001
40–44	5.453	0.119	5.220	5.687	< 0.001	5.259	0.157	4.951	5.568	< 0.001
45–49	8.763	0.122	8.524	9.002	< 0.001	8.311	0.152	8.013	8.610	< 0.001
50–54	8.329	0.125	8.083	8.575	< 0.001	7.782	0.158	7.473	8.091	< 0.001
55–59	10.700	0.123	10.459	10.940	< 0.001	10.171	0.154	9.87	10.472	< 0.001
60–64	9.476	0.134	9.213	9.739	< 0.001	9.010	0.171	8.675	9.345	< 0.001
65–69	11.203	0.139	10.930	11.476	< 0.001	10.589	0.175	10.246	10.933	< 0.001
70–74	11.546	0.171	11.210	11.882	< 0.001	11.255	0.194	10.876	11.635	< 0.001
75–79	13.387	0.200	12.995	13.779	< 0.001	13.086	0.212	12.67	13.503	< 0.001
**Sex (%)**										
Male	Refer					Refer				
Female	2.53	0.074	2.384	2.676	< 0.001	2.584	0.093	2.401	2.767	< 0.001
**History of CVD (%)**										
No	Refer					Refer				
Yes	0.085	0.064	− 0.041	0.212	0.186	0.184	0.083	0.021	0.348	0.027
**History of diabetes (%)**										
No	Refer					Refer				
Yes	0.075	0.079	− 0.078	0.229	0.661	− 0.02	0.097	− 0.21	0.169	0.834
**History of hyperlipidemia (%)**										
No	Refer					Refer				
Yes	0.652	0.123	0.411	0.892	0.892	0.716	0.151	0.419	1.013	< 0.001
**BMI group (%)**										
18.5–25	Refer					Refer				
< 18.5	1.149	0.166	0.824	1.474	< 0.001	1.052	0.225	0.610	1.494	< 0.001
25–30	2.317	0.172	1.980	2.654	< 0.001	1.868	0.234	1.409	2.327	< 0.001
> 30	3.065	0.213	2.647	3.483	< 0.001	2.208	0.281	1.657	2.760	< 0.001
**High risk drinking (%)**										
No	Refer					Refer				
Yes	− 0.205	0.108	− 0.416	0.006	0.057	− 0.186	0.139	− 0.459	0.086	0.180
**Physical activity (%)**										
Not regular	Refer					Refer				
Regular	− 0.096	0.070	− 0.232	0.041	0.170	0.014	0.104	− 0.189	0.218	0.889
**Diabetes (%)**										
No	Refer					Refer				
Yes	− 0.215	0.122	− 0.453	0.023	0.077	0.320	0.151	0.024	0.615	0.034

Parameters	Lead (μg/dL)					Mercury (μg/L)				
	ß	SE	95% CI		p-value	ß	SE	95% CI		p-value
		Adjusted R^2^ = 0.773, p < 0.001					Adjusted R^2^ = 0.773, p < 0.001			
*(B) For lead and mercury*										
Serum lead or mercury	0.104	0.056	0.016	0.214	0.044	0.110	0.036	0.039	0.181	0.003
Serum cotinine (ng/mL)	0.0002	0.00005	0.0001	0.0003	< 0.001	0.0002	0.00005	0.0001	0.0003	< 0.001
**Age group (%)**										
20–34	Refer					Refer				Refer
35–39	5.376	0.113	5.154	5.598	< 0.001	5.365	0.113	5.143	5.586	< 0.001
40–44	5.509	0.117	5.280	5.738	< 0.001	5.502	0.116	5.275	5.730	< 0.001
45–49	8.837	0.117	8.607	9.067	< 0.001	8.836	0.116	8.608	9.064	< 0.001
50–54	8.406	0.121	8.169	8.643	< 0.001	8.409	0.118	8.177	8.642	< 0.001
55–59	10.765	0.120	10.531	11.000	< 0.001	10.782	0.116	10.556	11.009	< 0.001
60–64	9.555	0.130	9.301	9.810	< 0.001	9.577	0.126	9.330	9.824	< 0.001
65–69	11.288	0.134	11.025	11.551	< 0.001	11.322	0.13	11.067	11.578	< 0.001
70–74	11.630	0.167	11.302	11.958	< 0.001	11.671	0.164	11.349	11.993	< 0.001
75–79	13.484	0.195	13.101	13.867	< 0.001	13.524	0.193	13.146	13.903	< 0.001
**Sex (%)**										
Male	Refer					Refer				Refer
female	2.641	0.074	2.496	2.787	< 0.001	2.661	0.074	2.517	2.805	< 0.001
**History of CVD (%)**										
No	Refer					Refer				
Yes	0.087	0.065	− 0.039	0.213	0.177	0.083	0.064	− 0.043	0.209	0.199
**History of diabetes (%)**										
No	Refer					Refer				
Yes	0.075	0.079	− 0.079	0.229	0.339	0.070	0.079	− 0.083	0.224	0.369
**History of hyperlipidemia (%)**										
No	Refer					Refer				Refer
Yes	0.656	0.123	0.416	0.897	< 0.001	0.657	0.123	0.417	0.897	< 0.001
**BMI group (%)**										
18.5–25	Refer					Refer				
< 18.5	1.142	.166	.816	1.467	< 0.001	1.134	0.166	0.808	1.459	< 0.001
25–30	2.315	.172	1.978	2.653	< 0.001	2.292	0.172	1.954	2.630	< 0.001
> 30	3.069	.213	2.65	3.487	< 0.001	3.028	0.214	2.609	3.447	< 0.001
**High risk drinking (%)**										
No	Refer					Refer				
Yes	− 0.221	0.108	− 0.433	− 0.009	0.041	− 0.228	0.108	− 0.440	− 0.017	0.035
**Physical activity (%)**										
Not regular	Refer					Refer				
Regular	− 0.101	0.070	− 0.238	0.036	0.147	− 0.101	0.070	− 0.238	0.036	0.147
**Diabetes (%)**										
No	Refer					Refer				
Yes	− 0.216	0.122	− 0.454	0.023	0.076	− 0.210	0.122	− 0.448	0.028	0.084

**Figure 3:**
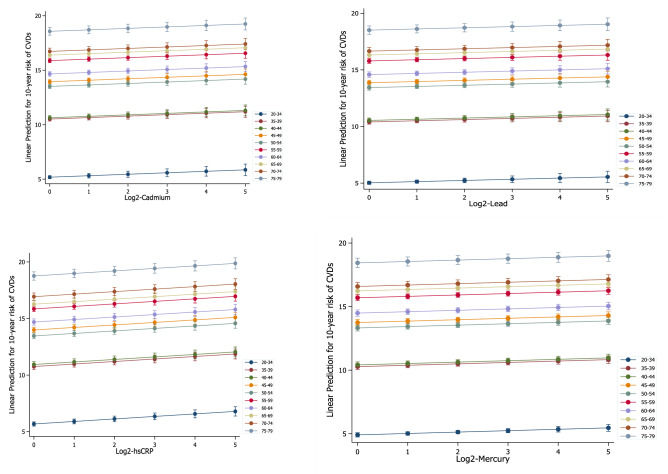
The marginal effect of the levels of serum lead, cadmium, mercury and hs-CRP on the 10-year risk of CVD by age group among the Korean population.

Figure 3 shows the marginal effect of the levels of serum heavy metals, and hs-CRP on the 10-year risk of CVD by age group after adjustment for potential confounders among the Korean population. The effect of heavy metals and hs-CRP showed a similar trend. An increase in serum cadmium, lead, mercury and hs-CRP was associated with an increase in the 10-year risk of CVD in each age group.

## Discussion

Our findings include empirical data that continues to draw on an important volume of previous studies to support the association between heavy metals and the 10-year risk of CVD among adult Koreans at the national level. More specifically, an increase in serum levels of cadmium, lead, mercury or CRP was associated with an increase in the 10-year risk of CVD.

The strong positive association found in our study between lead and 10-year risk of CVD emphasizes lead exposure as an important public health problem and concern^18^. The mediation of accelerated systolic blood pressure and renal damage is two main mechanisms through which lead has been involved in the risk of CVDs^19^. Besides, another hypothesis showed that the association of lead with atherosclerosis due to lead-induced oxidative stress and inflammation^20^. On the other hand, several studies have also reported on the association between exposure to cadmium or lead and elevated blood pressure. It could be explained that lead exposure may alter the renin-angiotensin system and cause disorders of sodium balance, raise vasoconstrictor prostaglandins, lower vasodilator prostaglandins^21^. Additionally, chronic lead exposure causes hypertension and CVDs by impairing nitric oxide signaling, reducing endothelium-dependent vasorelaxation, and altering the vascular response to vasoactive agonists^22,23^.

We found that there was a positive association between levels of cadmium and the 10-year risk of CVD. The adverse effects of cadmium on the vascular system are attributed to being mediated by inflammation, oxidative stress, and endothelial cell damage, which could lead to atherosclerosis^24^. Furthermore, Cadmium may cause CVDs through its adverse effects on the kidney due to nephrotoxicity and direct vasoconstriction^25,26^. Serum cadmium levels are also a positive correlation with hypertension^27^. On the other hand, oxidative stress induced by exposure to cadmium or lead, causes DNA damage and oxidizes protein thiol groups^20^. Cadmium or lead may also destroy blood clotting and provoke the production of inflammatory cytokines and anti-thrombotic agents^5,6^.

The exact biological mechanisms by which mercury produces toxic effects on CVDs remain unclear. However, our findings show higher serum mercury levels are associated with a significantly higher risk of CVD development, which is consistent with previous studies^28,29^. It could be explained that mercury exposure may increase the formation of ROS, free radicals, ROS, and superoxide anions, and reduce antioxidant enzyme activity (e.g., glutathione peroxidase, catalase, and superoxide dismutase), which can cause an increased risk of developing CVDs^30^. Taken together, these mechanisms support our results about the association between serum cadmium, lead, mercury levels and 10-year risk of CVD.

Our findings show that the association of aging with an increase in serum cadmium and lead; serum cotinine levels were positively correlated with serum cadmium and lead, which was in line with the previous studies^27,31,32^. It partly explained why strong significant correlations were pointed out between the 10-year risk of CVD and age. Of note, an increase in serum cadmium and lead levels was associated with an increase in serum cotinine levels, which was in agreement with the previous study^32^.

Interestingly, we also found that the effect of heavy metals (cadmium, lead and mercury) on lipid metabolism, especially total cholesterol and LDL-C, which concurs with the previous studies. For example, Kristal-Boneh et al. showed that the subjects exposed to lead had higher serum total cholesterol levels compared to those that did not^33^. Cho et al. also found a strong link between mercury exposure, LDL-C levels, and total cholesterol in the Korean general population^34^. In vivo studies also showed that an increase in serum cadmium levels was associated with increased levels of serum total cholesterol, LDL-C and triglyceride, and reduced HDL-C and glutathione levels^35,36^. Several mechanisms have been proposed to explain their associations with total cholesterol. First, lead may enhance hepatic gene expression of lanosterol 14α-demethylase (CYP51), which is a cytochrome P450 isoform, causing an increase in cellular cholesterol and total cholesterol levels^37^. Furthermore, the suppression of catabolic enzymes (e.g., 7 alpha-hydroxylase) and the activation of cholesterol synthesis enzymes (e.g., farnesyl diphosphate synthase, 3-hydroxyl-3-methylglutaryl-CoA reductase, squalene synthase) were associated with lead-mediated hypercholesterolemia^37^. Second, mercury may increase lipid peroxidation, serum oxidized LDL, and oxidation of LDL. These processes make metabolism of LDL difficult and result in its subsequent accumulation^38^. Third, Cadmium may deplete protein-bound sulfhydryl groups and glutathione, which leads to an increase in the production of ROS such as hydroxyl radicals, superoxide ion, and hydrogen peroxide. These ROS are known to induce increased excretion of urinary lipid metabolites and lipid peroxidation^39^.

hs-CRP is an independent risk factor for CVDs^40^. Another study found that hs-CRP levels above 10 mg/L are associated with a greater than 4% risk of developing a fatal CVD in 10 years^41^. Another study also reported the hs-CRP level above 10 mg/L is related to an over 4 percent risk of developing a fatal CVD in 10 years^42^. In several aspects of atherogenesis, hs-CRP plays a fundamental role, including the macrophage lipid uptake, release of proinflammatory cytokines, activation of the complement pathway, promotes endothelial dysfunction, induces tissue factor expression in monocytes and inhibits the development of nitric oxide^43^. These findings support our results that an increase in serum levels of hs-CRP was associated with an increase in the 10-year risk of CVDs.

Heavy metals such as cadmium, lead or mercury are toxic to the human body and can trigger different diseases, especially CVDs^44^. As a result, the prevalence of CVDs and exposure to heavy metal in Korea tends to be increasing^45–47^, these have worsened during the COVID-19 pandemic^48^. Unfortunately, the dramatic global increase in urbanization and industrialization has increased the risk of exposure to heavy metals^32^. For example, cadmium is abundant in groundwater and common foods such as rice, vegetables^24^. Remarkably, serum cadmium, lead and mercury levels are appropriate biomarkers for recent exposures to lead and cadmium^49,50^. Therefore, special concern should be given to the harmful impacts of heavy metals on the 10-year risk of CVDs. It is important to develop a prevention strategy targeting the high-risk population to slow down this progression to risk factors related to heavy metals and reduce prevalence. hs-CRP is the most validated and widely used inflammatory marker, and could be a potential clinical value in predicting and monitoring CVDs.

This large-scale Korean study is to report the effect of heavy metals on the 10-year risk of CVD at a national level. However, it has several limitations. First, the cross-sectional method used prevented evaluation of causality between 10-year risk of CVD and serum heavy metals. Second, actual CVD events (e.g., stroke, coronary heart disease or heart failure) were not evaluated. Third, the levels of heavy metals in the whole blood were not measured.

## Methods

### Study population

The heavy metal dataset of the Korean National Health and Nutrition Examination Survey (KNHANES) IV (2009), KNHANES V (2010–2012), KNHANES VI (2013), and KNHANES VII (2016–2017)^51^, a representative annual survey of the blood heavy metal concentrations, health, and nutritional status in the civilian, non-institutionalized Korean general population, was used. A total of 10,533 (2009), 8958 (2010), 8518 (2011), 8058 (2011), 8018 (2013), 8150 (2016), and 8127 (2017) subjects participated in the KNHANES. Of the 60,362 participants who underwent the survey from 2009–2013 to 2016–2017, we excluded 14,369 subjects less than 20 years old, 159 subjects more than 80 years old, 31,286 records missing serum Pb, Cd, missing laboratory test results [total cholesterol (1), HDL (3), systolic blood pressure (34), cotinine (4879)], and information on hypertension treatment (30). Consequently, a total of 9602 were eligible for data analysis. All participants in KNHANES provided written informed consent before examinations, which were performed by the Health and Nutrition Examination Department of the Korea Centers for Disease Control and Prevention. This study was approved by the KNHANES inquiry commission (IRB Approval numbers: 2009-01CON-03-2C, 2010-02CON-21-C, 2011-02CON-06-C, 2012-01EXP-01-2C, 2013-07CON-03-4C, 2013-12EXP-03-5C). From 2016 to 2017, KNHANES was exempt from review regarding research ethics under the Bioethics and Safety Act.

### Determination of serum Pb, and Cd levels

Pb, Hg, and Cd levels in serum were measured as previously described^9^. Serum cadmium, lead and mercury concentrations were determined by the NEODIN Medical Institute, certified by the Ministry of Health and Welfare of Korea. These tests meet the requirements of the German External Quality Assessment Scheme, the U.S. CDC, and the Korea Occupational Safety and Health Administration program. cadmium and lead were measured by graphite furnace atomic absorption spectrometry (model AAnalyst 600; Perkin Elmer, Turku, Finland) using Zeeman background correction. Total mercury was measured using a direct mercury analyzer (model DMA-80 Analyzer; Bergamo, Italy). Limits of detection (LODs) for lead, mercury, and cadmium were 0.223 µg/dL, 0.05 µg/L, and 0.087 µg/L, respectively. No sample had a value of below a LOD. For internal quality assurance and control, commercial standards (Lyphochek Whole Blood Metals, Bio-Rad, CA, USA) were used as reference materials.

### Urinary cotinine and smoking verification

Spot urinary samples were collected for a quantity of urinary cotinine by gas chromatography and mass spectrometry using PerkinElmer Clarus 600 T, with a detection limit of 1.26 ng/ml. Standard reference materials have been used for internal quality assurance and control purposes (ClinChek, RECIPE, Munich, Germany). The G-EQUAS uses a standard protocol to measure urinary cotinine. Subjects with urinary cotinine ≥ 50 ng/mL were defined as cotinine-verified smokers^52,53^.

### Laboratory measurements

Information on age, education, smoking history, and alcohol intake was collected during medical checkups using the standard procedure. Height and weight measurements were performed with the participants wearing light clothing and no shoes. Body mass index (BMI) was calculated as weight in kilograms divided by the square of the height in meters. Waist circumstance (cm) was measured at the midpoint between the bottom of the rib cage and the iliac crest of the mid-axillary line when exhaling. Blood pressure was measured with the participants in a seated position following a 5-min rest period. Blood pressure was measured in the right arm on three occasions using a mercury sphygmomanometer and was averaged to determine the final blood pressure reading. Blood samples were collected in the morning after an overnight fast. Serum concentrations of high-density lipoprotein cholesterol (HDL-C), triglycerides, alanine aspartate aminotransferase (ALT), aspartate aminotransferase (AST), and glucose were measured using an automatic analyzer (Hitachi 7600; Hitachi, Tokyo, Japan). Serum low-density lipoprotein cholesterol (LDL-C) was calculated using the Friedewald equation: serum LDL-C ¼ serum total cholesterol–serum HDL-C-serum triglyceride/5^3^. hs-CRP) level was measured with immunoturbidimetry using the Cobas 8000 (Roche, Mannheim, Germany). All clinical analyses were performed by the Neodin Medical Institute, a laboratory certified by the Korean Ministry of Health and Welfare.

### Parameters

Alcohol intakes were categorized as low or high-risk drinking (high-risk drinking was defined as > 5 drinks per day and ≥ 1 month). Physical activity has been dichotomized as regular or irregular. Regular physical activity was defined as: (1) participation in vigorous physical activity (running, fast cycling, climbing, football, fast swimming, basketball, squash, singles tennis, rope jumping or occupational or recreational activity involving the carrying of heavy objects), ≥ 20 min per session ≥ 3 days per week (2) or participation in moderate physical activity (slow swimming, volleyball, doubles tennis, or occupational or recreational activity involving the carrying of light objects); ≥ 30 min per session ≥ 5 days per week; (3) or participation in walking; ≥ 30 min per session ≥ 5 days per week^54^.

### Assessment of nutrient intake

All participants were required to maintain their usual dietary habits before collecting data on dietary intake. Daily food intake was measured using the 24-h recall method, and nutrient intake was calculated using the Can-Pro 3.0 nutrient intake assessment software developed by the Korean Nutrition Society^3^.

### Framingham estimate of 10-year coronary heart disease (CVD) risk

The Framingham risk equation was used for the estimation of 10-year CVD risk for each participant. The Framingham estimate of 10-year risk of CVD was derived from the Framingham point score, based on HDL cholesterol, total cholesterol concentrations, age, systolic blood pressure, and smoking by gender. The total risk factors ranged from 0–17 in males and 1–25 in females, representing Framingham point scores ranging from 1 to 30%^17^. They are categorized as low risk, < 10%; intermediate-risk, 10%–19%; and high risk, ≥ 20%^55^.

### Statistical analysis

All statistical analyses were undertaken using STATA software (version 16.0; StataCorp, Texas, USA). The baseline characteristics of participants were summarized using frequency and proportion for categorical variables; mean and standard deviation for continuous variables.

Pearson’s correlation coefficient was calculated for checking the relationships between levels of serum heavy metals and cardiometabolic risk factors, dietary intake. To define different levels of serum cadmium, lead, mercury, and hs-CRP, we categorized them into quartiles. We compared the mean values of cardiometabolic risk factors according to the quartiles of serum cadmium, lead, mercury, and hs-CRP using ANOVA (one-way) OR or Mann–Whitney test was performed independently for each variable.

The serum heavy metals (cadmium, lead, mercury, and hs-CRP) levels were log_2_-transformed because their distribution was right skewed. The serum heavy metal levels were described as the geometric mean (GM) and 95% confidence interval (CI).

A multiple regression analysis was used to analyze the associations between the blood heavy metal levels and 10-year risk of CVDs. The regression analyses were adjusted for serum cotinine (*ng/mL*), age group (20–34, 35–39, 40–44, 45–49, 50–59, 60–64, 65–69, 70–74, 75–79), sex (males, females), high-risk drinking (yes, no), physical activity (not regular, regular), BMI groups (< 18.5, ≥ 18.5 and < 25, ≥ 25 and < 30, ≥ 30), family history of CVDs, or diabetes or dyslipidemia (yes, no), and type 2 diabetes. The marginal effects were then used to predict the 10-year risk of CVD. Statistical tests were two-sided, *p*-value < 0.05 was considered statistically significant.
